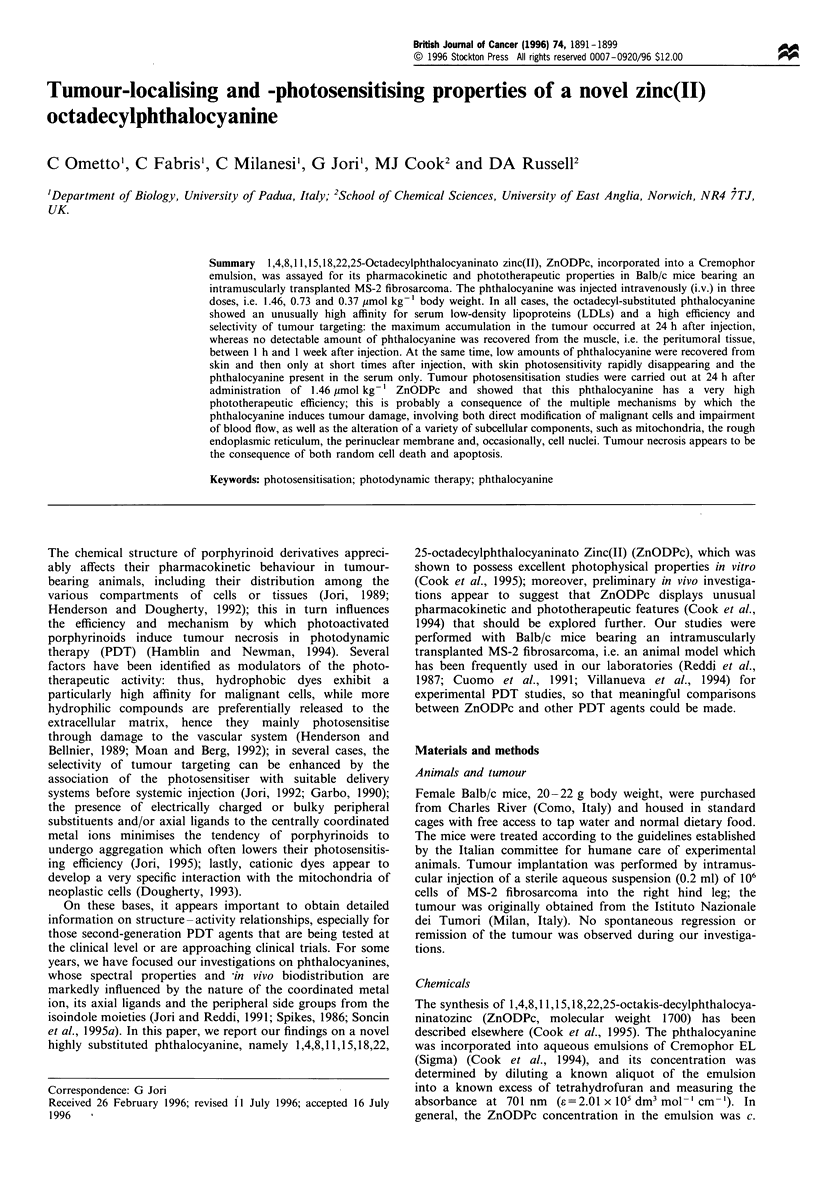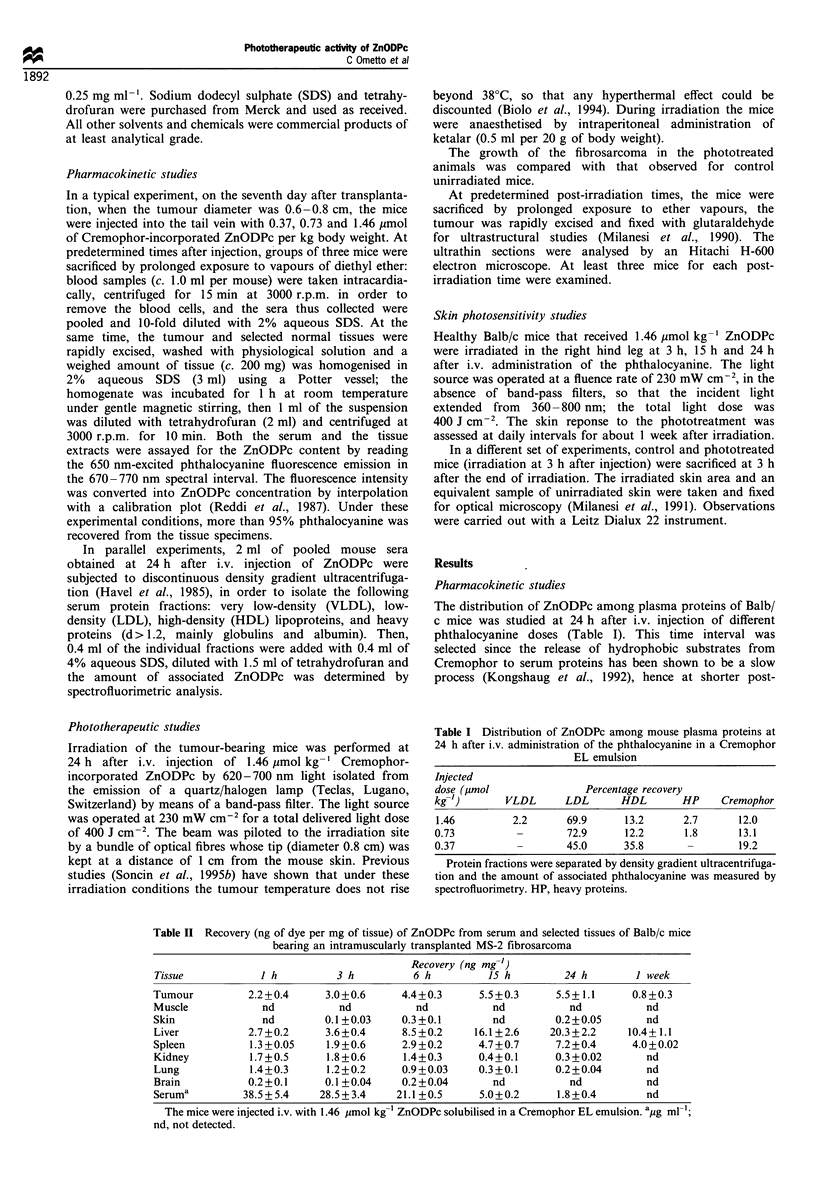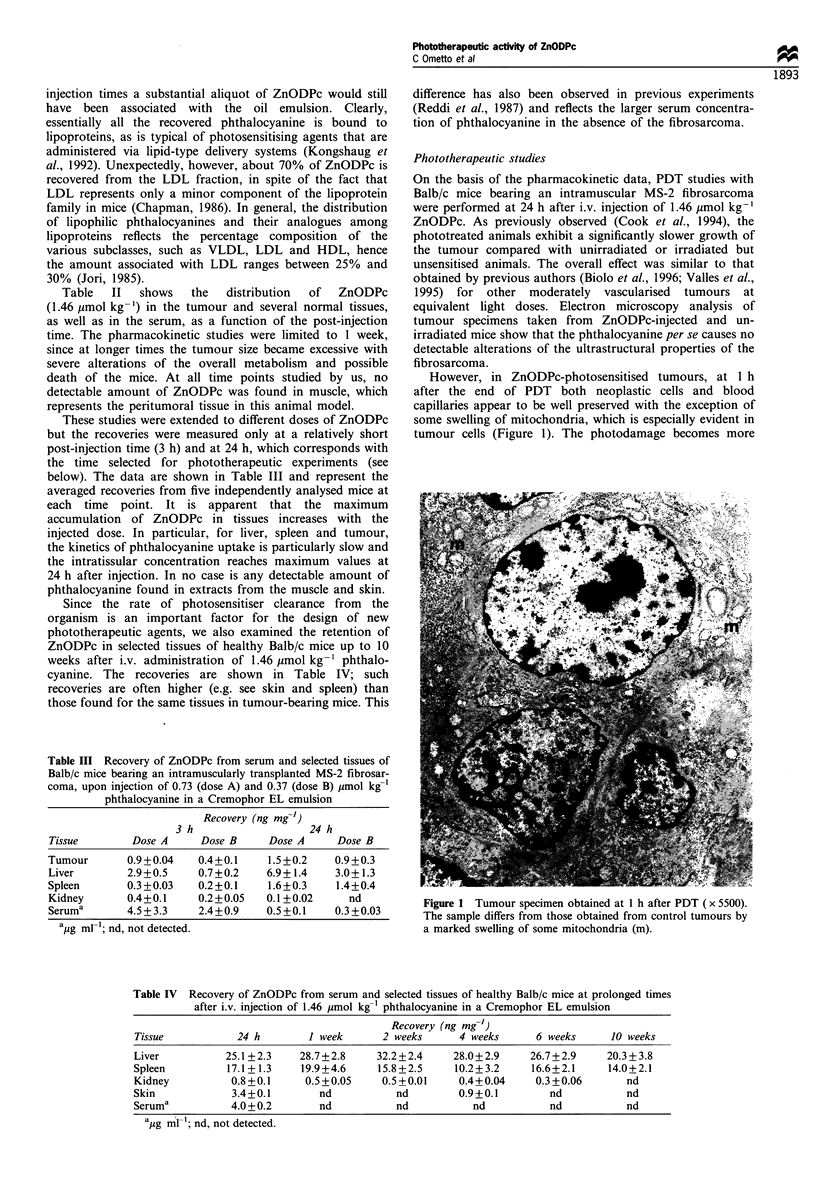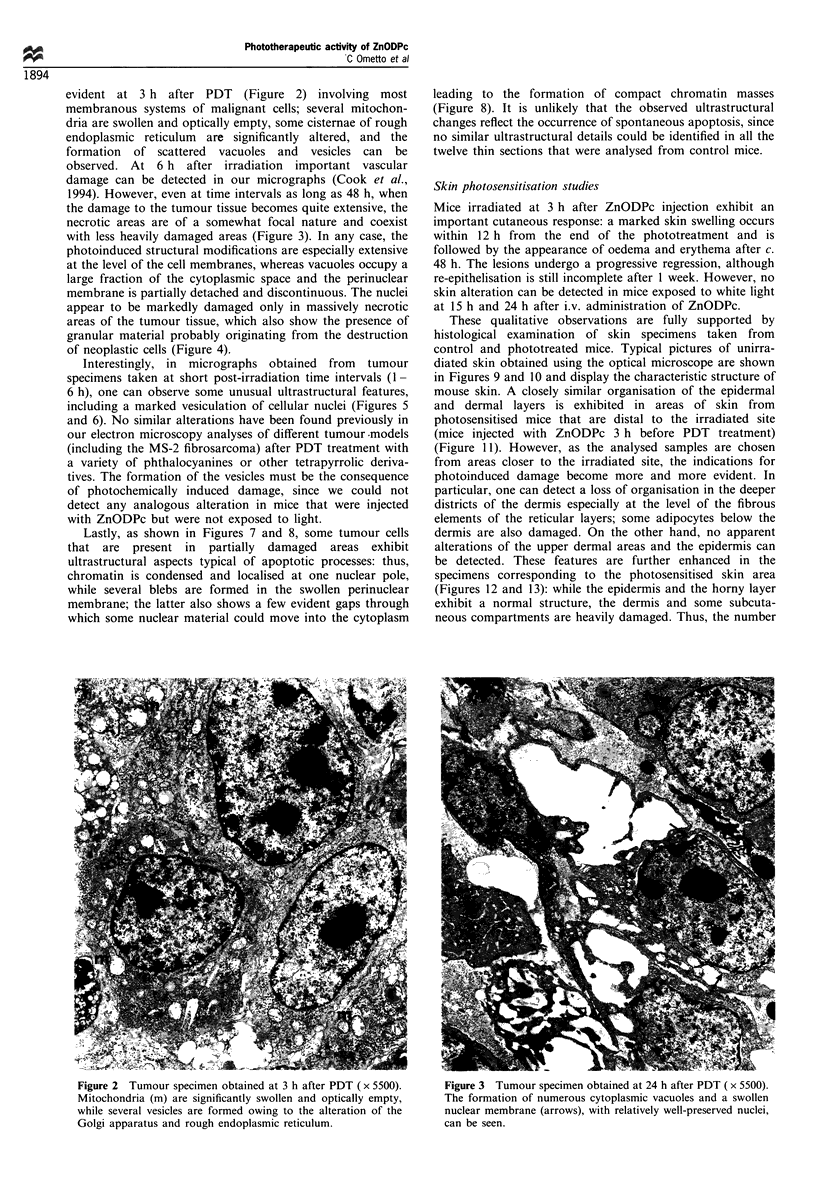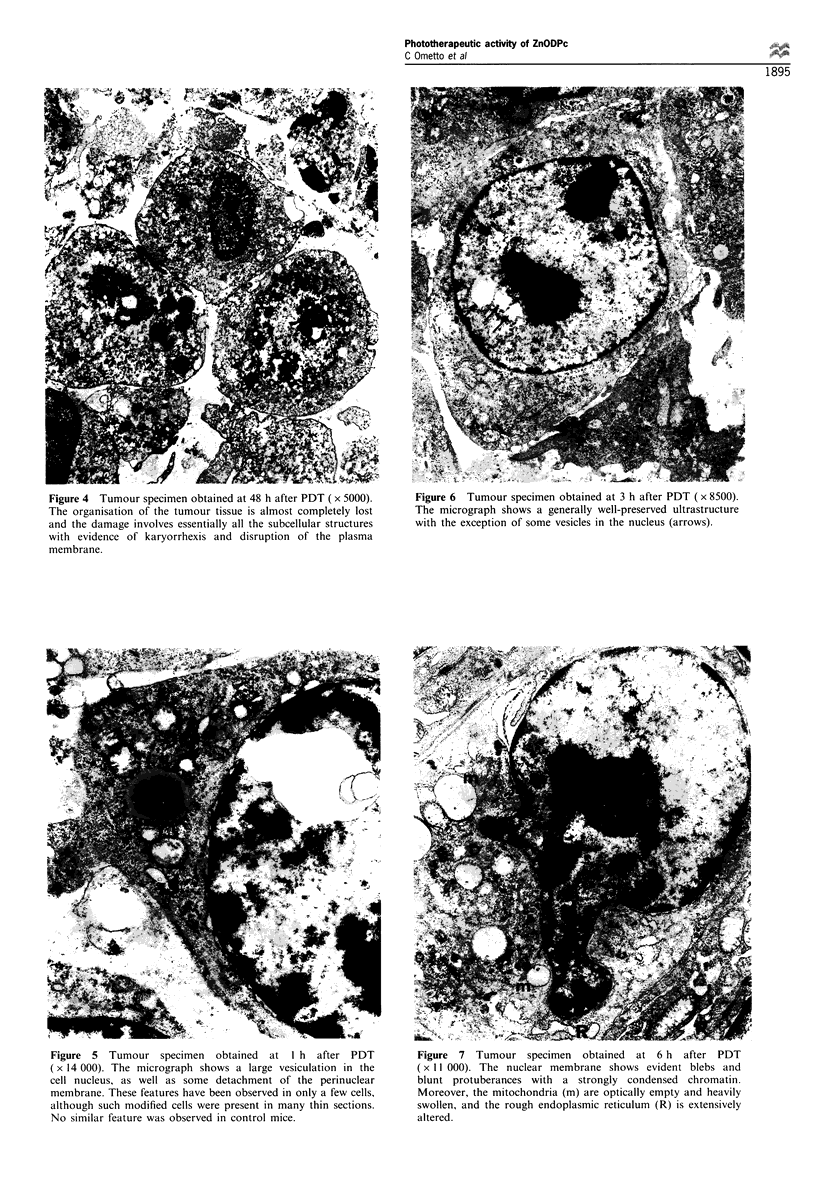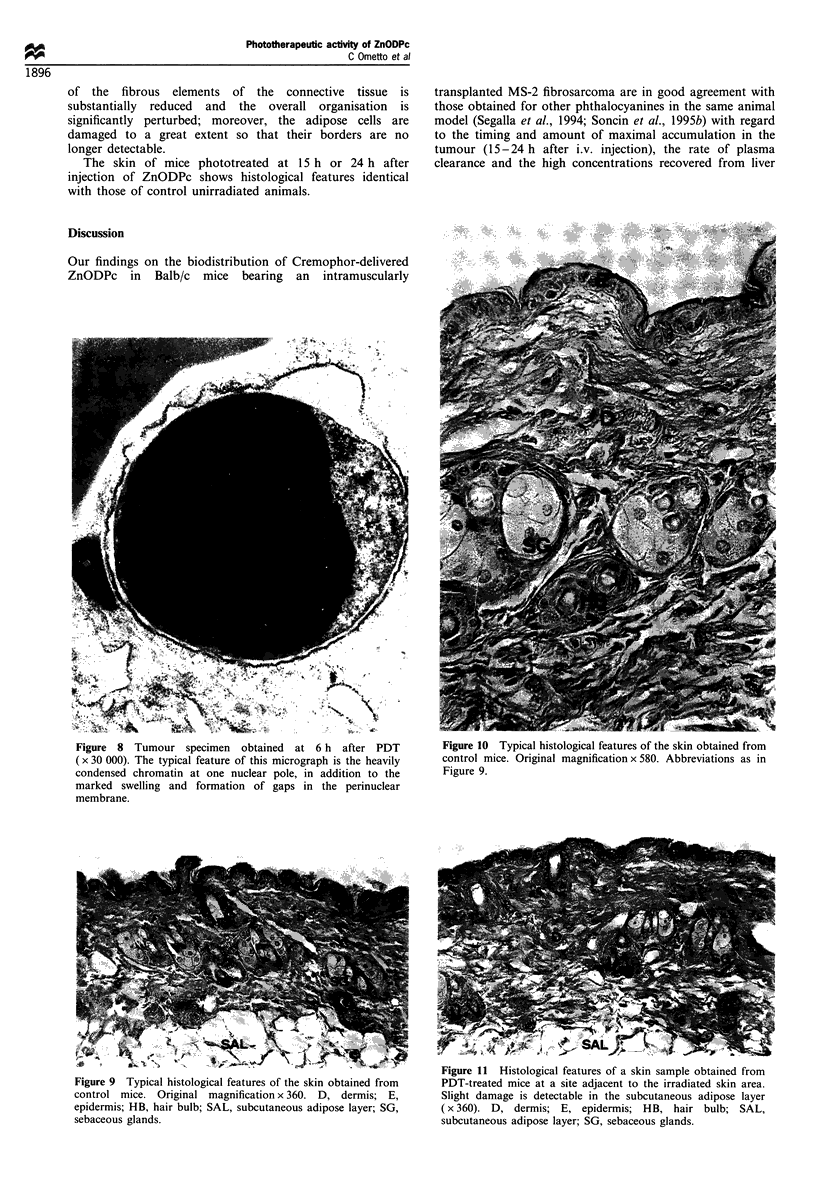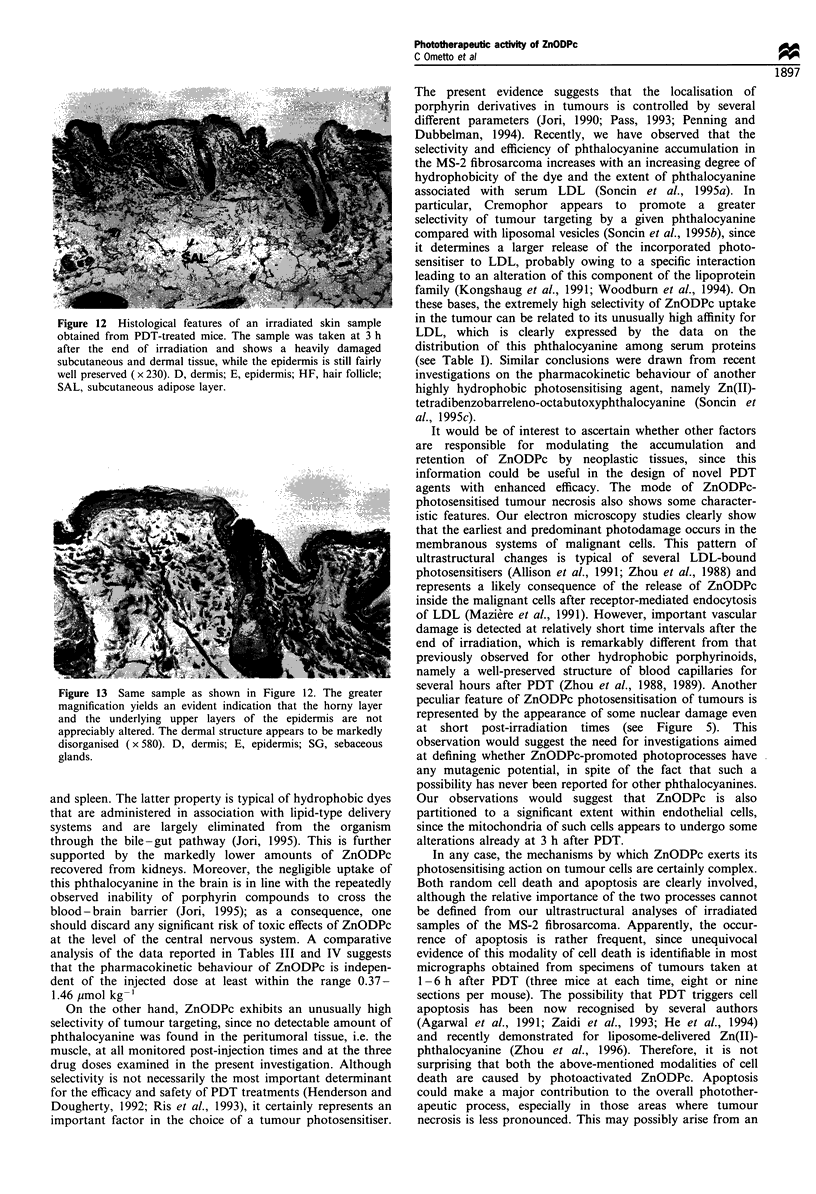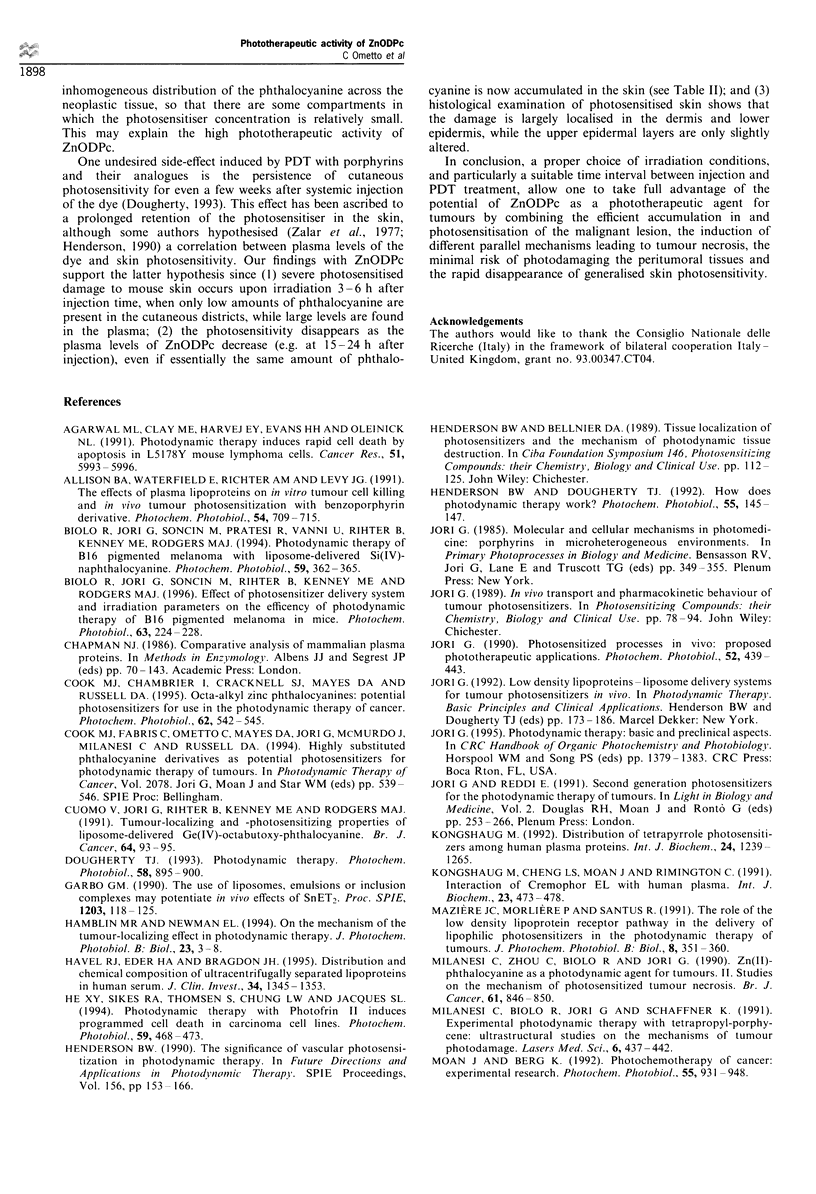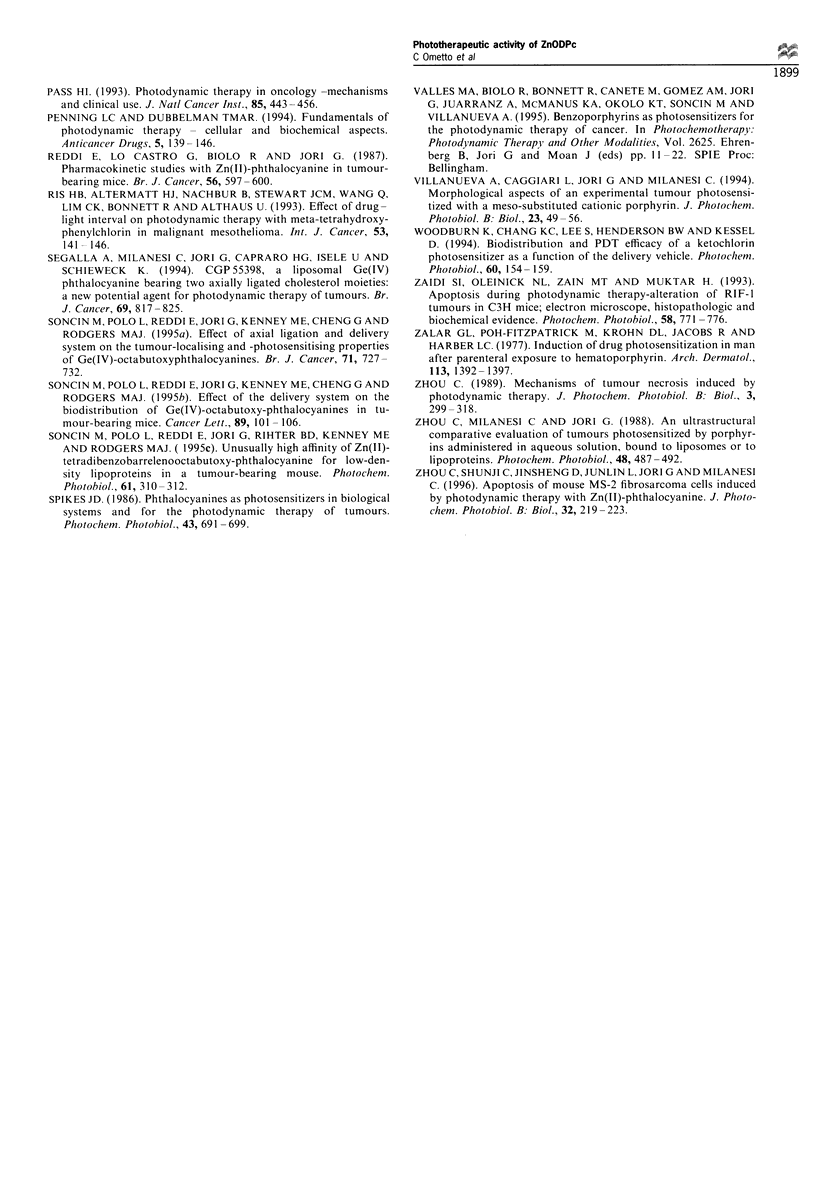# Tumour-localising and -photosensitising properties of a novel zinc(II) octadecylphthalocyanine.

**DOI:** 10.1038/bjc.1996.650

**Published:** 1996-12

**Authors:** C. Ometto, C. Fabris, C. Milanesi, G. Jori, M. J. Cook, D. A. Russell

**Affiliations:** Department of Biology, University of Padua, Italy.

## Abstract

**Images:**


British Journal of Cancer (1996) 74, 1891-1899
© 1996 Stockton Press All rights reserved 0007-0920/96 $12.00

### Tumour-localising and -photosensitising properties of a novel zinc(II) octadecylphthalocyanine

C Ometto[1], C Fabris[1], C Milanesi[1], G Jori[1], MJ Cook[2] and DA Russell[2]

[1]*Department of Biology, University of Padua, Italy;* [2]*School of Chemical Sciences, University of East Anglia, Norwich, NR4 7TJ, UK.*

**Summary** 1,4,8,11,15,18,22,25-Octadecylphthalocyaninato zinc(II), ZnODPc, incorporated into a Cremophor emulsion, was assayed for its pharmacokinetic and phototherapeutic properties in Balb/c mice bearing an intramuscularly transplanted MS-2 fibrosarcoma. The phthalocyanine was injected intravenously (i.v.) in three doses, i.e. 1.46, 0.73 and 0.37 $\mu$mol kg$^{-1}$ body weight. In all cases, the octadecyl-substituted phthalocyanine showed an unusually high affinity for serum low-density lipoproteins (LDLs) and a high efficiency and selectivity of tumour targeting: the maximum accumulation in the tumour occurred at 24 h after injection, whereas no detectable amount of phthalocyanine was recovered from the muscle, i.e. the peritumoral tissue, between 1 h and 1 week after injection. At the same time, low amounts of phthalocyanine were recovered from skin and then only at short times after injection, with skin photosensitivity rapidly disappearing and the phthalocyanine present in the serum only. Tumour photosensitisation studies were carried out at 24 h after administration of 1.46 $\mu$mol kg$^{-1}$ ZnODPc and showed that this phthalocyanine has a very high phototherapeutic efficiency; this is probably a consequence of the multiple mechanisms by which the phthalocyanine induces tumour damage, involving both direct modification of malignant cells and impairment of blood flow, as well as the alteration of a variety of subcellular components, such as mitochondria, the rough endoplasmic reticulum, the perinuclear membrane and, occasionally, cell nuclei. Tumour necrosis appears to be the consequence of both random cell death and apoptosis.

**Keywords:** photosensitisation; photodynamic therapy; phthalocyanine

The chemical structure of porphyrinoid derivatives appreciably affects their pharmacokinetic behaviour in tumour-bearing animals, including their distribution among the various compartments of cells or tissues (Jori, 1989; Henderson and Dougherty, 1992); this in turn influences the efficiency and mechanism by which photoactivated porphyrinoids induce tumour necrosis in photodynamic therapy (PDT) (Hamblin and Newman, 1994). Several factors have been identified as modulators of the phototherapeutic activity: thus, hydrophobic dyes exhibit a particularly high affinity for malignant cells, while more hydrophilic compounds are preferentially released to the extracellular matrix, hence they mainly photosensitise through damage to the vascular system (Henderson and Bellnier, 1989; Moan and Berg, 1992); in several cases, the selectivity of tumour targeting can be enhanced by the association of the photosensitiser with suitable delivery systems before systemic injection (Jori, 1992; Garbo, 1990); the presence of electrically charged or bulky peripheral substituents and/or axial ligands to the centrally coordinated metal ions minimises the tendency of porphyrinoids to undergo aggregation which often lowers their photosensitising efficiency (Jori, 1995); lastly, cationic dyes appear to develop a very specific interaction with the mitochondria of neoplastic cells (Dougherty, 1993).

On these bases, it appears important to obtain detailed information on structure–activity relationships, especially for those second-generation PDT agents that are being tested at the clinical level or are approaching clinical trials. For some years, we have focused our investigations on phthalocyanines, whose spectral properties and *in vivo* biodistribution are markedly influenced by the nature of the coordinated metal ion, its axial ligands and the peripheral side groups from the isoindole moieties (Jori and Reddi, 1991; Spikes, 1986; Soncin *et al.*, 1995*a*). In this paper, we report our findings on a novel highly substituted phthalocyanine, namely 1,4,8,11,15,18,22,25-octadecylphthalocyaninato Zinc(II) (ZnODPc), which was shown to possess excellent photophysical properties *in vitro* (Cook *et al.*, 1995); moreover, preliminary *in vivo* investigations appear to suggest that ZnODPc displays unusual pharmacokinetic and phototherapeutic features (Cook *et al.*, 1994) that should be explored further. Our studies were performed with Balb/c mice bearing an intramuscularly transplanted MS-2 fibrosarcoma, i.e. an animal model which has been frequently used in our laboratories (Reddi *et al.*, 1987; Cuomo *et al.*, 1991; Villanueva *et al.*, 1994) for experimental PDT studies, so that meaningful comparisons between ZnODPc and other PDT agents could be made.

### Materials and methods

### *Animals and tumour*

Female Balb/c mice, 20–22 g body weight, were purchased from Charles River (Como, Italy) and housed in standard cages with free access to tap water and normal dietary food. The mice were treated according to the guidelines established by the Italian committee for humane care of experimental animals. Tumour implantation was performed by intramuscular injection of a sterile aqueous suspension (0.2 ml) of $10^6$ cells of MS-2 fibrosarcoma into the right hind leg; the tumour was originally obtained from the Istituto Nazionale dei Tumori (Milan, Italy). No spontaneous regression or remission of the tumour was observed during our investigations.

### *Chemicals*

The synthesis of 1,4,8,11,15,18,22,25-octakis-decylphthalocyaninatozinc (ZnODPc, molecular weight 1700) has been described elsewhere (Cook *et al.*, 1995). The phthalocyanine was incorporated into aqueous emulsions of Cremophor EL (Sigma) (Cook *et al.*, 1994), and its concentration was determined by diluting a known aliquot of the emulsion into a known excess of tetrahydrofuran and measuring the absorbance at 701 nm ($\varepsilon = 2.01 \times 10^5$ dm$^3$ mol$^{-1}$ cm$^{-1}$). In general, the ZnODPc concentration in the emulsion was *c.*

Correspondence: G Jori
Received 26 February 1996; revised 11 July 1996; accepted 16 July 1996

0.25 mg ml$^{-1}$. Sodium dodecyl sulphate (SDS) and tetrahydrofuran were purchased from Merck and used as received. All other solvents and chemicals were commercial products of at least analytical grade.

*Pharmacokinetic studies*

In a typical experiment, on the seventh day after transplantation, when the tumour diameter was 0.6–0.8 cm, the mice were injected into the tail vein with 0.37, 0.73 and 1.46 $\mu$mol of Cremophor-incorporated ZnODPc per kg body weight. At predetermined times after injection, groups of three mice were sacrificed by prolonged exposure to vapours of diethyl ether: blood samples (*c*. 1.0 ml per mouse) were taken intracardiacally, centrifuged for 15 min at 3000 r.p.m. in order to remove the blood cells, and the sera thus collected were pooled and 10-fold diluted with 2% aqueous SDS. At the same time, the tumour and selected normal tissues were rapidly excised, washed with physiological solution and a weighed amount of tissue (*c*. 200 mg) was homogenised in 2% aqueous SDS (3 ml) using a Potter vessel; the homogenate was incubated for 1 h at room temperature under gentle magnetic stirring, then 1 ml of the suspension was diluted with tetrahydrofuran (2 ml) and centrifuged at 3000 r.p.m. for 10 min. Both the serum and the tissue extracts were assayed for the ZnODPc content by reading the 650 nm-excited phthalocyanine fluorescence emission in the 670–770 nm spectral interval. The fluorescence intensity was converted into ZnODPc concentration by interpolation with a calibration plot (Reddi *et al*., 1987). Under these experimental conditions, more than 95% phthalocyanine was recovered from the tissue specimens.

In parallel experiments, 2 ml of pooled mouse sera obtained at 24 h after i.v. injection of ZnODPc were subjected to discontinuous density gradient ultracentrifugation (Havel *et al*., 1985), in order to isolate the following serum protein fractions: very low-density (VLDL), low-density (LDL), high-density (HDL) lipoproteins, and heavy proteins (d > 1.2, mainly globulins and albumin). Then, 0.4 ml of the individual fractions were added with 0.4 ml of 4% aqueous SDS, diluted with 1.5 ml of tetrahydrofuran and the amount of associated ZnODPc was determined by spectrofluorimetric analysis.

*Phototherapeutic studies*

Irradiation of the tumour-bearing mice was performed at 24 h after i.v. injection of 1.46 $\mu$mol kg$^{-1}$ Cremophor-incorporated ZnODPc by 620–700 nm light isolated from the emission of a quartz/halogen lamp (Teclas, Lugano, Switzerland) by means of a band-pass filter. The light source was operated at 230 mW cm$^{-2}$ for a total delivered light dose of 400 J cm$^{-2}$. The beam was piloted to the irradiation site by a bundle of optical fibres whose tip (diameter 0.8 cm) was kept at a distance of 1 cm from the mouse skin. Previous studies (Soncin *et al*., 1995*b*) have shown that under these irradiation conditions the tumour temperature does not rise beyond 38°C, so that any hyperthermal effect could be discounted (Biolo *et al*., 1994). During irradiation the mice were anaesthetised by intraperitoneal administration of ketalar (0.5 ml per 20 g of body weight).

The growth of the fibrosarcoma in the phototreated animals was compared with that observed for control unirradiated mice.

At predetermined post-irradiation times, the mice were sacrificed by prolonged exposure to ether vapours, the tumour was rapidly excised and fixed with glutaraldehyde for ultrastructural studies (Milanesi *et al*., 1990). The ultrathin sections were analysed by an Hitachi H-600 electron microscope. At least three mice for each post-irradiation time were examined.

*Skin photosensitivity studies*

Healthy Balb/c mice that received 1.46 $\mu$mol kg$^{-1}$ ZnODPc were irradiated in the right hind leg at 3 h, 15 h and 24 h after i.v. administration of the phthalocyanine. The light source was operated at a fluence rate of 230 mW cm$^{-2}$, in the absence of band-pass filters, so that the incident light extended from 360–800 nm; the total light dose was 400 J cm$^{-2}$. The skin reponse to the phototreatment was assessed at daily intervals for about 1 week after irradiation.

In a different set of experiments, control and phototreated mice (irradiation at 3 h after injection) were sacrificed at 3 h after the end of irradiation. The irradiated skin area and an equivalent sample of unirradiated skin were taken and fixed for optical microscopy (Milanesi *et al*., 1991). Observations were carried out with a Leitz Dialux 22 instrument.

### Results

*Pharmacokinetic studies*

The distribution of ZnODPc among plasma proteins of Balb/c mice was studied at 24 h after i.v. injection of different phthalocyanine doses (Table I). This time interval was selected since the release of hydrophobic substrates from Cremophor to serum proteins has been shown to be a slow process (Kongshaug *et al*., 1992), hence at shorter post-

**Table I** Distribution of ZnODPc among mouse plasma proteins at 24 h after i.v. administration of the phthalocyanine in a Cremophor EL emulsion

| *Injected dose (µmol kg$^{-1}$)* | *Percentage recovery* | | | | |
|---|---|---|---|---|---|
| | *VLDL* | *LDL* | *HDL* | *HP* | *Cremophor* |
| 1.46 | 2.2 | 69.9 | 13.2 | 2.7 | 12.0 |
| 0.73 | – | 72.9 | 12.2 | 1.8 | 13.1 |
| 0.37 | – | 45.0 | 35.8 | – | 19.2 |

Protein fractions were separated by density gradient ultracentrifugation and the amount of associated phthalocyanine was measured by spectrofluorimetry. HP, heavy proteins.

**Table II** Recovery (ng of dye per mg of tissue) of ZnODPc from serum and selected tissues of Balb/c mice bearing an intramuscularly transplanted MS-2 fibrosarcoma

| *Tissue* | *Recovery (ng mg$^{-1}$)* | | | | | |
|---|---|---|---|---|---|---|
| | *1 h* | *3 h* | *6 h* | *15 h* | *24 h* | *1 week* |
| Tumour | 2.2±0.4 | 3.0±0.6 | 4.4±0.3 | 5.5±0.3 | 5.5±1.1 | 0.8±0.3 |
| Muscle | nd | nd | nd | nd | nd | nd |
| Skin | nd | 0.1±0.03 | 0.3±0.1 | nd | 0.2±0.05 | nd |
| Liver | 2.7±0.2 | 3.6±0.4 | 8.5±0.2 | 16.1±2.6 | 20.3±2.2 | 10.4±1.1 |
| Spleen | 1.3±0.05 | 1.9±0.6 | 2.9±0.2 | 4.7±0.7 | 7.2±0.4 | 4.0±0.02 |
| Kidney | 1.7±0.5 | 1.8±0.6 | 1.4±0.3 | 0.4±0.1 | 0.3±0.02 | nd |
| Lung | 1.4±0.3 | 1.2±0.2 | 0.9±0.03 | 0.3±0.1 | 0.2±0.04 | nd |
| Brain | 0.2±0.1 | 0.1±0.04 | 0.2±0.04 | nd | nd | nd |
| Serum[a] | 38.5±5.4 | 28.5±3.4 | 21.1±0.5 | 5.0±0.2 | 1.8±0.4 | nd |

The mice were injected i.v. with 1.46 $\mu$mol kg$^{-1}$ ZnODPc solubilised in a Cremophor EL emulsion. [a]$\mu$g ml$^{-1}$; nd, not detected.

injection times a substantial aliquot of ZnODPc would still have been associated with the oil emulsion. Clearly, essentially all the recovered phthalocyanine is bound to lipoproteins, as is typical of photosensitising agents that are administered via lipid-type delivery systems (Kongshaug *et al.*, 1992). Unexpectedly, however, about 70% of ZnODPc is recovered from the LDL fraction, in spite of the fact that LDL represents only a minor component of the lipoprotein family in mice (Chapman, 1986). In general, the distribution of lipophilic phthalocyanines and their analogues among lipoproteins reflects the percentage composition of the various subclasses, such as VLDL, LDL and HDL, hence the amount associated with LDL ranges between 25% and 30% (Jori, 1985).

Table II shows the distribution of ZnODPc (1.46 $\mu$mol kg$^{-1}$) in the tumour and several normal tissues, as well as in the serum, as a function of the post-injection time. The pharmacokinetic studies were limited to 1 week, since at longer times the tumour size became excessive with severe alterations of the overall metabolism and possible death of the mice. At all time points studied by us, no detectable amount of ZnODPc was found in muscle, which represents the peritumoral tissue in this animal model.

These studies were extended to different doses of ZnODPc but the recoveries were measured only at a relatively short post-injection time (3 h) and at 24 h, which corresponds with the time selected for phototherapeutic experiments (see below). The data are shown in Table III and represent the averaged recoveries from five independently analysed mice at each time point. It is apparent that the maximum accumulation of ZnODPc in tissues increases with the injected dose. In particular, for liver, spleen and tumour, the kinetics of phthalocyanine uptake is particularly slow and the intratissular concentration reaches maximum values at 24 h after injection. In no case is any detectable amount of phthalocyanine found in extracts from the muscle and skin.

Since the rate of photosensitiser clearance from the organism is an important factor for the design of new phototherapeutic agents, we also examined the retention of ZnODPc in selected tissues of healthy Balb/c mice up to 10 weeks after i.v. administration of 1.46 $\mu$mol kg$^{-1}$ phthalocyanine. The recoveries are shown in Table IV; such recoveries are often higher (e.g. see skin and spleen) than those found for the same tissues in tumour-bearing mice. This difference has also been observed in previous experiments (Reddi *et al.*, 1987) and reflects the larger serum concentration of phthalocyanine in the absence of the fibrosarcoma.

*Phototherapeutic studies*

On the basis of the pharmacokinetic data, PDT studies with Balb/c mice bearing an intramuscular MS-2 fibrosarcoma were performed at 24 h after i.v. injection of 1.46 $\mu$mol kg$^{-1}$ ZnODPc. As previously observed (Cook *et al.*, 1994), the phototreated animals exhibit a significantly slower growth of the tumour compared with unirradiated or irradiated but unsensitised animals. The overall effect was similar to that obtained by previous authors (Biolo *et al.*, 1996; Valles *et al.*, 1995) for other moderately vascularised tumours at equivalent light doses. Electron microscopy analysis of tumour specimens taken from ZnODPc-injected and unirradiated mice show that the phthalocyanine *per se* causes no detectable alterations of the ultrastructural properties of the fibrosarcoma.

However, in ZnODPc-photosensitised tumours, at 1 h after the end of PDT both neoplastic cells and blood capillaries appear to be well preserved with the exception of some swelling of mitochondria, which is especially evident in tumour cells (Figure 1). The photodamage becomes more

**Table III** Recovery of ZnODPc from serum and selected tissues of Balb/c mice bearing an intramuscularly transplanted MS-2 fibrosarcoma, upon injection of 0.73 (dose A) and 0.37 (dose B) $\mu$mol kg$^{-1}$ phthalocyanine in a Cremophor EL emulsion

| | *Recovery (ng mg$^{-1}$)* | | | |
|---|---|---|---|---|
| | *3 h* | | *24 h* | |
| *Tissue* | *Dose A* | *Dose B* | *Dose A* | *Dose B* |
| Tumour | 0.9±0.04 | 0.4±0.1 | 1.5±0.2 | 0.9±0.3 |
| Liver | 2.9±0.5 | 0.7±0.2 | 6.9±1.4 | 3.0±1.3 |
| Spleen | 0.3±0.03 | 0.2±0.1 | 1.6±0.3 | 1.4±0.4 |
| Kidney | 0.4±0.1 | 0.2±0.05 | 0.1±0.02 | nd |
| Serum[a] | 4.5±3.3 | 2.4±0.9 | 0.5±0.1 | 0.3±0.03 |

[a]$\mu$g ml$^{-1}$; nd, not detected.

**Figure 1** Tumour specimen obtained at 1 h after PDT (×5500). The sample differs from those obtained from control tumours by a marked swelling of some mitochondria (m).

**Table IV** Recovery of ZnODPc from serum and selected tissues of healthy Balb/c mice at prolonged times after i.v. injection of 1.46 $\mu$mol kg$^{-1}$ phthalocyanine in a Cremophor EL emulsion

| | *Recovery (ng mg$^{-1}$)* | | | | | |
|---|---|---|---|---|---|---|
| *Tissue* | *24 h* | *1 week* | *2 weeks* | *4 weeks* | *6 weeks* | *10 weeks* |
| Liver | 25.1±2.3 | 28.7±2.8 | 32.2±2.4 | 28.0±2.9 | 26.7±2.9 | 20.3±3.8 |
| Spleen | 17.1±1.3 | 19.9±4.6 | 15.8±2.5 | 10.2±3.2 | 16.6±2.1 | 14.0±2.1 |
| Kidney | 0.8±0.1 | 0.5±0.05 | 0.5±0.01 | 0.4±0.04 | 0.3±0.06 | nd |
| Skin | 3.4±0.1 | nd | nd | 0.9±0.1 | nd | nd |
| Serum[a] | 4.0±0.2 | nd | nd | nd | nd | nd |

[a]$\mu$g ml$^{-1}$; nd, not detected.

evident at 3 h after PDT (Figure 2) involving most membranous systems of malignant cells; several mitochondria are swollen and optically empty, some cisternae of rough endoplasmic reticulum are significantly altered, and the formation of scattered vacuoles and vesicles can be observed. At 6 h after irradiation important vascular damage can be detected in our micrographs (Cook *et al.*, 1994). However, even at time intervals as long as 48 h, when the damage to the tumour tissue becomes quite extensive, the necrotic areas are of a somewhat focal nature and coexist with less heavily damaged areas (Figure 3). In any case, the photoinduced structural modifications are especially extensive at the level of the cell membranes, whereas vacuoles occupy a large fraction of the cytoplasmic space and the perinuclear membrane is partially detached and discontinuous. The nuclei appear to be markedly damaged only in massively necrotic areas of the tumour tissue, which also show the presence of granular material probably originating from the destruction of neoplastic cells (Figure 4).

Interestingly, in micrographs obtained from tumour specimens taken at short post-irradiation time intervals (1–6 h), one can observe some unusual ultrastructural features, including a marked vesiculation of cellular nuclei (Figures 5 and 6). No similar alterations have been found previously in our electron microscopy analyses of different tumour models (including the MS-2 fibrosarcoma) after PDT treatment with a variety of phthalocyanines or other tetrapyrrolic derivatives. The formation of the vesicles must be the consequence of photochemically induced damage, since we could not detect any analogous alteration in mice that were injected with ZnODPc but were not exposed to light.

Lastly, as shown in Figures 7 and 8, some tumour cells that are present in partially damaged areas exhibit ultrastructural aspects typical of apoptotic processes: thus, chromatin is condensed and localised at one nuclear pole, while several blebs are formed in the swollen perinuclear membrane; the latter also shows a few evident gaps through which some nuclear material could move into the cytoplasm leading to the formation of compact chromatin masses (Figure 8). It is unlikely that the observed ultrastructural changes reflect the occurrence of spontaneous apoptosis, since no similar ultrastructural details could be identified in all the twelve thin sections that were analysed from control mice.

*Skin photosensitisation studies*

Mice irradiated at 3 h after ZnODPc injection exhibit an important cutaneous response: a marked skin swelling occurs within 12 h from the end of the phototreatment and is followed by the appearance of oedema and erythema after *c.* 48 h. The lesions undergo a progressive regression, although re-epithelisation is still incomplete after 1 week. However, no skin alteration can be detected in mice exposed to white light at 15 h and 24 h after i.v. administration of ZnODPc.

These qualitative observations are fully supported by histological examination of skin specimens taken from control and phototreated mice. Typical pictures of unirradiated skin obtained using the optical microscope are shown in Figures 9 and 10 and display the characteristic structure of mouse skin. A closely similar organisation of the epidermal and dermal layers is exhibited in areas of skin from photosensitised mice that are distal to the irradiated site (mice injected with ZnODPc 3 h before PDT treatment) (Figure 11). However, as the analysed samples are chosen from areas closer to the irradiated site, the indications for photoinduced damage become more and more evident. In particular, one can detect a loss of organisation in the deeper districts of the dermis especially at the level of the fibrous elements of the reticular layers; some adipocytes below the dermis are also damaged. On the other hand, no apparent alterations of the upper dermal areas and the epidermis can be detected. These features are further enhanced in the specimens corresponding to the photosensitised skin area (Figures 12 and 13): while the epidermis and the horny layer exhibit a normal structure, the dermis and some subcutaneous compartments are heavily damaged. Thus, the number

**Figure 2** Tumour specimen obtained at 3 h after PDT (×5500). Mitochondria (m) are significantly swollen and optically empty, while several vesicles are formed owing to the alteration of the Golgi apparatus and rough endoplasmic reticulum.

**Figure 3** Tumour specimen obtained at 24 h after PDT (×5500). The formation of numerous cytoplasmic vacuoles and a swollen nuclear membrane (arrows), with relatively well-preserved nuclei, can be seen.

**Figure 4** Tumour specimen obtained at 48 h after PDT ( × 5000). The organisation of the tumour tissue is almost completely lost and the damage involves essentially all the subcellular structures with evidence of karyorrhexis and disruption of the plasma membrane.

**Figure 5** Tumour specimen obtained at 1 h after PDT ( × 14 000). The micrograph shows a large vesiculation in the cell nucleus, as well as some detachment of the perinuclear membrane. These features have been observed in only a few cells, although such modified cells were present in many thin sections. No similar feature was observed in control mice.

**Figure 6** Tumour specimen obtained at 3 h after PDT ( × 8500). The micrograph shows a generally well-preserved ultrastructure with the exception of some vesicles in the nucleus (arrows).

**Figure 7** Tumour specimen obtained at 6 h after PDT ( × 11 000). The nuclear membrane shows evident blebs and blunt protuberances with a strongly condensed chromatin. Moreover, the mitochondria (m) are optically empty and heavily swollen, and the rough endoplasmic reticulum (R) is extensively altered.

of the fibrous elements of the connective tissue is substantially reduced and the overall organisation is significantly perturbed; moreover, the adipose cells are damaged to a great extent so that their borders are no longer detectable.

The skin of mice phototreated at 15 h or 24 h after injection of ZnODPc shows histological features identical with those of control unirradiated animals.

### Discussion

Our findings on the biodistribution of Cremophor-delivered ZnODPc in Balb/c mice bearing an intramuscularly transplanted MS-2 fibrosarcoma are in good agreement with those obtained for other phthalocyanines in the same animal model (Segalla *et al*., 1994; Soncin *et al*., 1995*b*) with regard to the timing and amount of maximal accumulation in the tumour (15–24 h after i.v. injection), the rate of plasma clearance and the high concentrations recovered from liver

**Figure 8** Tumour specimen obtained at 6 h after PDT ( × 30 000). The typical feature of this micrograph is the heavily condensed chromatin at one nuclear pole, in addition to the marked swelling and formation of gaps in the perinuclear membrane.

**Figure 10** Typical histological features of the skin obtained from control mice. Original magnification × 580. Abbreviations as in Figure 9.

**Figure 9** Typical histological features of the skin obtained from control mice. Original magnification × 360. D, dermis; E, epidermis; HB, hair bulb; SAL, subcutaneous adipose layer; SG, sebaceous glands.

**Figure 11** Histological features of a skin sample obtained from PDT-treated mice at a site adjacent to the irradiated skin area. Slight damage is detectable in the subcutaneous adipose layer ( × 360). D, dermis; E, epidermis; HB, hair bulb; SAL, subcutaneous adipose layer; SG, sebaceous glands.

**Figure 12** Histological features of an irradiated skin sample obtained from PDT-treated mice. The sample was taken at 3 h after the end of irradiation and shows a heavily damaged subcutaneous and dermal tissue, while the epidermis is still fairly well preserved (×230). D, dermis; E, epidermis; HF, hair follicle; SAL, subcutaneous adipose layer.

**Figure 13** Same sample as shown in Figure 12. The greater magnification yields an evident indication that the horny layer and the underlying upper layers of the epidermis are not appreciably altered. The dermal structure appears to be markedly disorganised (×580). D, dermis; E, epidermis; SG, sebaceous glands.

and spleen. The latter property is typical of hydrophobic dyes that are administered in association with lipid-type delivery systems and are largely eliminated from the organism through the bile–gut pathway (Jori, 1995). This is further supported by the markedly lower amounts of ZnODPc recovered from kidneys. Moreover, the negligible uptake of this phthalocyanine in the brain is in line with the repeatedly observed inability of porphyrin compounds to cross the blood–brain barrier (Jori, 1995); as a consequence, one should discard any significant risk of toxic effects of ZnODPc at the level of the central nervous system. A comparative analysis of the data reported in Tables III and IV suggests that the pharmacokinetic behaviour of ZnODPc is independent of the injected dose at least within the range 0.37–1.46 $\mu$mol $kg^{-1}$

On the other hand, ZnODPc exhibits an unusually high selectivity of tumour targeting, since no detectable amount of phthalocyanine was found in the peritumoral tissue, i.e. the muscle, at all monitored post-injection times and at the three drug doses examined in the present investigation. Although selectivity is not necessarily the most important determinant for the efficacy and safety of PDT treatments (Henderson and Dougherty, 1992; Ris *et al.*, 1993), it certainly represents an important factor in the choice of a tumour photosensitiser. The present evidence suggests that the localisation of porphyrin derivatives in tumours is controlled by several different parameters (Jori, 1990; Pass, 1993; Penning and Dubbelman, 1994). Recently, we have observed that the selectivity and efficiency of phthalocyanine accumulation in the MS-2 fibrosarcoma increases with an increasing degree of hydrophobicity of the dye and the extent of phthalocyanine associated with serum LDL (Soncin *et al.*, 1995*a*). In particular, Cremophor appears to promote a greater selectivity of tumour targeting by a given phthalocyanine compared with liposomal vesicles (Soncin *et al.*, 1995*b*), since it determines a larger release of the incorporated photosensitiser to LDL, probably owing to a specific interaction leading to an alteration of this component of the lipoprotein family (Kongshaug *et al.*, 1991; Woodburn *et al.*, 1994). On these bases, the extremely high selectivity of ZnODPc uptake in the tumour can be related to its unusually high affinity for LDL, which is clearly expressed by the data on the distribution of this phthalocyanine among serum proteins (see Table I). Similar conclusions were drawn from recent investigations on the pharmacokinetic behaviour of another highly hydrophobic photosensitising agent, namely Zn(II)-tetradibenzobarreleno-octabutoxyphthalocyanine (Soncin *et al.*, 1995*c*).

It would be of interest to ascertain whether other factors are responsible for modulating the accumulation and retention of ZnODPc by neoplastic tissues, since this information could be useful in the design of novel PDT agents with enhanced efficacy. The mode of ZnODPc-photosensitised tumour necrosis also shows some characteristic features. Our electron microscopy studies clearly show that the earliest and predominant photodamage occurs in the membranous systems of malignant cells. This pattern of ultrastructural changes is typical of several LDL-bound photosensitisers (Allison *et al.*, 1991; Zhou *et al.*, 1988) and represents a likely consequence of the release of ZnODPc inside the malignant cells after receptor-mediated endocytosis of LDL (Mazière *et al.*, 1991). However, important vascular damage is detected at relatively short time intervals after the end of irradiation, which is remarkably different from that previously observed for other hydrophobic porphyrinoids, namely a well-preserved structure of blood capillaries for several hours after PDT (Zhou *et al.*, 1988, 1989). Another peculiar feature of ZnODPc photosensitisation of tumours is represented by the appearance of some nuclear damage even at short post-irradiation times (see Figure 5). This observation would suggest the need for investigations aimed at defining whether ZnODPc-promoted photoprocesses have any mutagenic potential, in spite of the fact that such a possibility has never been reported for other phthalocyanines. Our observations would suggest that ZnODPc is also partitioned to a significant extent within endothelial cells, since the mitochondria of such cells appears to undergo some alterations already at 3 h after PDT.

In any case, the mechanisms by which ZnODPc exerts its photosensitising action on tumour cells are certainly complex. Both random cell death and apoptosis are clearly involved, although the relative importance of the two processes cannot be defined from our ultrastructural analyses of irradiated samples of the MS-2 fibrosarcoma. Apparently, the occurrence of apoptosis is rather frequent, since unequivocal evidence of this modality of cell death is identifiable in most micrographs obtained from specimens of tumours taken at 1–6 h after PDT (three mice at each time, eight or nine sections per mouse). The possibility that PDT triggers cell apoptosis has been now recognised by several authors (Agarwal *et al.*, 1991; Zaidi *et al.*, 1993; He *et al.*, 1994) and recently demonstrated for liposome-delivered Zn(II)-phthalocyanine (Zhou *et al.*, 1996). Therefore, it is not surprising that both the above-mentioned modalities of cell death are caused by photoactivated ZnODPc. Apoptosis could make a major contribution to the overall phototherapeutic process, especially in those areas where tumour necrosis is less pronounced. This may possibly arise from an inhomogeneous distribution of the phthalocyanine across the neoplastic tissue, so that there are some compartments in which the photosensitiser concentration is relatively small. This may explain the high phototherapeutic activity of ZnODPc.

One undesired side-effect induced by PDT with porphyrins and their analogues is the persistence of cutaneous photosensitivity for even a few weeks after systemic injection of the dye (Dougherty, 1993). This effect has been ascribed to a prolonged retention of the photosensitiser in the skin, although some authors hypothesised (Zalar *et al.*, 1977; Henderson, 1990) a correlation between plasma levels of the dye and skin photosensitivity. Our findings with ZnODPc support the latter hypothesis since (1) severe photosensitised damage to mouse skin occurs upon irradiation 3–6 h after injection time, when only low amounts of phthalocyanine are present in the cutaneous districts, while large levels are found in the plasma; (2) the photosensitivity disappears as the plasma levels of ZnODPc decrease (e.g. at 15–24 h after injection), even if essentially the same amount of phthalocyanine is now accumulated in the skin (see Table II); and (3) histological examination of photosensitised skin shows that the damage is largely localised in the dermis and lower epidermis, while the upper epidermal layers are only slightly altered.

In conclusion, a proper choice of irradiation conditions, and particularly a suitable time interval between injection and PDT treatment, allow one to take full advantage of the potential of ZnODPc as a phototherapeutic agent for tumours by combining the efficient accumulation in and photosensitisation of the malignant lesion, the induction of different parallel mechanisms leading to tumour necrosis, the minimal risk of photodamaging the peritumoral tissues and the rapid disappearance of generalised skin photosensitivity.

**Acknowledgements**
The authors would like to thank the Consiglio Nationale delle Ricerche (Italy) in the framework of bilateral cooperation Italy–United Kingdom, grant no. 93.00347.CT04.

### References

AGARWAL ML, CLAY ME, HARVEJ EY, EVANS HH AND OLEINICK NL. (1991). Photodynamic therapy induces rapid cell death by apoptosis in L5178Y mouse lymphoma cells. *Cancer Res.*, **51,** 5993–5996.

ALLISON BA, WATERFIELD E, RICHTER AM AND LEVY JG. (1991). The effects of plasma lipoproteins on *in vitro* tumour cell killing and *in vivo* tumour photosensitization with benzoporphyrin derivative. *Photochem. Photobiol.*, **54,** 709–715.

BIOLO R, JORI G, SONCIN M, PRATESI R, VANNI U, RIHTER B, KENNEY ME, RODGERS MAJ. (1994). Photodynamic therapy of B16 pigmented melanoma with liposome-delivered Si(IV)-naphthalocyanine. *Photochem. Photobiol.*, **59,** 362–365.

BIOLO R, JORI G, SONCIN M, RIHTER B, KENNEY ME AND RODGERS MAJ. (1996). Effect of photosensitizer delivery system and irradiation parameters on the efficency of photodynamic therapy of B16 pigmented melanoma in mice. *Photochem. Photobiol.*, **63,** 224–228.

CHAPMAN NJ. (1986). Comparative analysis of mammalian plasma proteins. In *Methods in Enzymology*. Albens JJ and Segrest JP (eds) pp. 70–143. Academic Press: London.

COOK MJ, CHAMBRIER I, CRACKNELL SJ, MAYES DA AND RUSSELL DA. (1995). Octa-alkyl zinc phthalocyanines: potential photosensitizers for use in the photodynamic therapy of cancer. *Photochem. Photobiol.*, **62,** 542–545.

COOK MJ, FABRIS C, OMETTO C, MAYES DA, JORI G, MCMURDO J, MILANESI C AND RUSSELL DA. (1994). Highly substituted phthalocyanine derivatives as potential photosensitizers for photodynamic therapy of tumours. In *Photodynamic Therapy of Cancer*, Vol. 2078. Jori G, Moan J and Star WM (eds) pp. 539–546. SPIE Proc: Bellingham.

CUOMO V, JORI G, RIHTER B, KENNEY ME AND RODGERS MAJ. (1991). Tumour-localizing and -photosensitizing properties of liposome-delivered Ge(IV)-octabutoxy-phthalocyanine. *Br. J. Cancer*, **64,** 93–95.

DOUGHERTY TJ. (1993). Photodynamic therapy. *Photochem. Photobiol.*, **58,** 895–900.

GARBO GM. (1990). The use of liposomes, emulsions or inclusion complexes may potentiate *in vivo* effects of $SnET_2$. *Proc. SPIE*, **1203,** 118–125.

HAMBLIN MR AND NEWMAN EL. (1994). On the mechanism of the tumour-localizing effect in photodynamic therapy. *J. Photochem. Photobiol. B: Biol.*, **23,** 3–8.

HAVEL RJ, EDER HA AND BRAGDON JH. (1995). Distribution and chemical composition of ultracentrifugally separated lipoproteins in human serum. *J. Clin. Invest.*, **34,** 1345–1353.

HE XY, SIKES RA, THOMSEN S, CHUNG LW AND JACQUES SL. (1994). Photodynamic therapy with Photofrin II induces programmed cell death in carcinoma cell lines. *Photochem. Photobiol.*, **59,** 468–473.

HENDERSON BW. (1990). The significance of vascular photosensitization in photodynamic therapy. In *Future Directions and Applications in Photodynomic Therapy*. SPIE Proceedings, Vol. 156, pp 153–166.

HENDERSON BW AND BELLNIER DA. (1989). Tissue localization of photosensitizers and the mechanism of photodynamic tissue destruction. In *Ciba Foundation Symposium 146, Photosensitizing Compounds: their Chemistry, Biology and Clinical Use*. pp. 112–125. John Wiley: Chichester.

HENDERSON BW AND DOUGHERTY TJ. (1992). How does photodynamic therapy work? *Photochem. Photobiol.*, **55,** 145–147.

JORI G. (1985). Molecular and cellular mechanisms in photomedicine: porphyrins in microheterogeneous environments. In *Primary Photoprocesses in Biology and Medicine*. Bensasson RV, Jori G, Lane E and Truscott TG (eds) pp. 349–355. Plenum Press: New York.

JORI G. (1989). *In vivo* transport and pharmacokinetic behaviour of tumour photosensitizers. In *Photosensitizing Compounds: their Chemistry, Biology and Clinical Use*. pp. 78–94. John Wiley: Chichester.

JORI G. (1990). Photosensitized processes in vivo: proposed phototherapeutic applications. *Photochem. Photobiol.*, **52,** 439–443.

JORI G. (1992). Low density lipoproteins–liposome delivery systems for tumour photosensitizers *in vivo*. In *Photodynamic Therapy. Basic Principles and Clinical Applications*. Henderson BW and Dougherty TJ (eds) pp. 173–186. Marcel Dekker: New York.

JORI G. (1995). Photodynamic therapy: basic and preclinical aspects. In *CRC Handbook of Organic Photochemistry and Photobiology*. Horspool WM and Song PS (eds) pp. 1379–1383. CRC Press: Boca Rton, FL, USA.

JORI G AND REDDI E. (1991). Second generation photosensitizers for the photodynamic therapy of tumours. In *Light in Biology and Medicine*, Vol. 2. Douglas RH, Moan J and Rontò G (eds) pp. 253–266, Plenum Press: London.

KONGSHAUG M. (1992). Distribution of tetrapyrrole photosensitizers among human plasma proteins. *Int. J. Biochem.*, **24,** 1239–1265.

KONGSHAUG M, CHENG LS, MOAN J AND RIMINGTON C. (1991). Interaction of Cremophor EL with human plasma. *Int. J. Biochem.*, **23,** 473–478.

MAZIÈRE JC, MORLIÈRE P AND SANTUS R. (1991). The role of the low density lipoprotein receptor pathway in the delivery of lipophilic photosensitizers in the photodynamic therapy of tumours. *J. Photochem. Photobiol. B: Biol.*, **8,** 351–360.

MILANESI C, ZHOU C, BIOLO R AND JORI G. (1990). Zn(II)-phthalocyanine as a photodynamic agent for tumours. II. Studies on the mechanism of photosensitized tumour necrosis. *Br. J. Cancer*, **61,** 846–850.

MILANESI C, BIOLO R, JORI G AND SCHAFFNER K. (1991). Experimental photodynamic therapy with tetrapropyl-porphycene: ultrastructural studies on the mechanisms of tumour photodamage. *Lasers Med. Sci.*, **6,** 437–442.

MOAN J AND BERG K. (1992). Photochemotherapy of cancer: experimental research. *Photochem. Photobiol.*, **55,** 931–948.

PASS HI. (1993). Photodynamic therapy in oncology -mechanisms and clinical use. *J. Natl Cancer Inst.*, **85**, 443-456.
PENNING LC AND DUBBELMAN TMAR. (1994). Fundamentals of photodynamic therapy - cellular and biochemical aspects. *Anticancer Drugs*, **5**, 139-146.
REDDI E, LO CASTRO G, BIOLO R AND JORI G. (1987). Pharmacokinetic studies with Zn(II)-phthalocyanine in tumour-bearing mice. *Br. J. Cancer*, **56**, 597-600.
RIS HB, ALTERMATT HJ, NACHBUR B, STEWART JCM, WANG Q, LIM CK, BONNETT R AND ALTHAUS U. (1993). Effect of drug-light interval on photodynamic therapy with meta-tetrahydroxyphenylchlorin in malignant mesothelioma. *Int. J. Cancer*, **53**, 141-146.
SEGALLA A, MILANESI C, JORI G, CAPRARO HG, ISELE U AND SCHIEWECK K. (1994). CGP 55398, a liposomal Ge(IV) phthalocyanine bearing two axially ligated cholesterol moieties: a new potential agent for photodynamic therapy of tumours. *Br. J. Cancer*, **69**, 817-825.
SONCIN M, POLO L, REDDI E, JORI G, KENNEY ME, CHENG G AND RODGERS MAJ. (1995*a*). Effect of axial ligation and delivery system on the tumour-localising and -photosensitising properties of Ge(IV)-octabutoxyphthalocyanines. *Br. J. Cancer*, **71**, 727-732.
SONCIN M, POLO L, REDDI E, JORI G, KENNEY ME, CHENG G AND RODGERS MAJ. (1995*b*). Effect of the delivery system on the biodistribution of Ge(IV)-octabutoxy-phthalocyanines in tumour-bearing mice. *Cancer Lett.*, **89**, 101-106.
SONCIN M, POLO L, REDDI E, JORI G, RIHTER BD, KENNEY ME AND RODGERS MAJ. ( 1995c). Unusually high affinity of Zn(II)-tetradibenzobarrelenooctabutoxy-phthalocyanine for low-density lipoproteins in a tumour-bearing mouse. *Photochem. Photobiol.*, **61**, 310-312.
SPIKES JD. (1986). Phthalocyanines as photosensitizers in biological systems and for the photodynamic therapy of tumours. *Photochem. Photobiol.*, **43**, 691-699.
VALLES MA, BIOLO R, BONNETT R, CANETE M, GOMEZ AM, JORI G, JUARRANZ A, MCMANUS KA, OKOLO KT, SONCIN M AND VILLANUEVA A. (1995). Benzoporphyrins as photosensitizers for the photodynamic therapy of cancer. In *Photochemotherapy: Photodynamic Therapy and Other Modalities*, Vol. 2625. Ehrenberg B, Jori G and Moan J (eds) pp. 11-22. SPIE Proc: Bellingham.
VILLANUEVA A, CAGGIARI L, JORI G AND MILANESI C. (1994). Morphological aspects of an experimental tumour photosensitized with a meso-substituted cationic porphyrin. *J. Photochem. Photobiol. B: Biol.*, **23**, 49-56.
WOODBURN K, CHANG KC, LEE S, HENDERSON BW AND KESSEL D. (1994). Biodistribution and PDT efficacy of a ketochlorin photosensitizer as a function of the delivery vehicle. *Photochem. Photobiol.*, **60**, 154-159.
ZAIDI SI, OLEINICK NL, ZAIN MT AND MUKTAR H. (1993). Apoptosis during photodynamic therapy-alteration of RIF-1 tumours in C3H mice; electron microscope, histopathologic and biochemical evidence. *Photochem. Photobiol.*, **58**, 771-776.
ZALAR GL, POH-FITZPATRICK M, KROHN DL, JACOBS R AND HARBER LC. (1977). Induction of drug photosensitization in man after parenteral exposure to hematoporphyrin. *Arch. Dermatol.*, **113**, 1392-1397.
ZHOU C. (1989). Mechanisms of tumour necrosis induced by photodynamic therapy. *J. Photochem. Photobiol. B: Biol.*, **3**, 299-318.
ZHOU C, MILANESI C AND JORI G. (1988). An ultrastructural comparative evaluation of tumours photosensitized by porphyrins administered in aqueous solution, bound to liposomes or to lipoproteins. *Photochem. Photobiol.*, **48**, 487-492.
ZHOU C, SHUNJI C, JINSHENG D, JUNLIN L, JORI G AND MILANESI C. (1996). Apoptosis of mouse MS-2 fibrosarcoma cells induced by photodynamic therapy with Zn(II)-phthalocyanine. *J. Photochem. Photobiol. B: Biol.*, **32**, 219-223.